# Comparative electrochemical study of veterinary drug danofloxacin at glassy carbon electrode and electrified liquid–liquid interface

**DOI:** 10.1038/s41598-024-65246-3

**Published:** 2024-06-24

**Authors:** Konrad Rudnicki, Sylwia Budzyńska, Sławomira Skrzypek, Lukasz Poltorak

**Affiliations:** 1 Department of Inorganic and Analytical Chemistry, Electrochemistry@Soft Interface Team, Faculty of Chemistry, University of Lodz, Tamka 12, 91-403 Łódź, Poland; 2 Department of Chemistry, Faculty of Forestry and Wood Technology, Poznań University of Life Sciences, Wojska Polskiego 75, 60-625 Poznań, Poland

**Keywords:** Danofloxacin, Fluoroquinolone antibiotic, Milk sample, Liquid–liquid interface, Glassy carbon electrode, Analytical chemistry, Biochemistry, Electrochemistry, Surface chemistry, Chemistry

## Abstract

This work compares the electroanalytical performance of two electroanalytical systems based on (1) the glassy carbon electrode (GCE), and (2) the electrified liquid–liquid interface (eLLI), for the detection of fluoroquinolone antibiotic–danofloxacin (DANO). Our aim was to define the optimal conditions to detect the chosen analyte with two employed systems, extract a number of electroanalytical parameters, study the mechanism of the charge transfer reactions (oxidation at GCE and ion transfer across the eLLI), and to provide physicochemical constants for DANO. Detection of the chosen analyte was also performed in the spiked milk samples. To the best of our knowledge, this is the first work that directly compares the electroanalytical parameters obtained with solid electrode (in this case GCE) and eLLI. We have found that for DANO the latter provides better electroanalytical parameters (lower LOD and LOQ) as well as good selectivity when the milk was analyzed.

## Introduction

Fluoroquinolone antibiotics (FQs) are a class of synthetic antimicrobial agents with a fluorine atom present within their chemical structure, typically attached to a quinolone ring. They possess potent antibacterial activity and have been widely used in clinical medicine to combat a broad spectrum of bacterial infections^1^. FQs are appreciated for their broad spectrum of action. As such, these molecules are frequently employed in the treatment of various infections, such as respiratory tract, urinary tract, skin and soft tissues, bones, and joints bacterial infections, among others. In recent years, FQs as a family of antimicrobial drugs gained a lot of scientific attention. This is due to reports of rare but serious side effects, including prolonged neurological and musculoskeletal issues, leading to stricter regulatory actions^2^. Consequently, their utilization in clinical practice is now strictly monitored.

**Figure 1:**
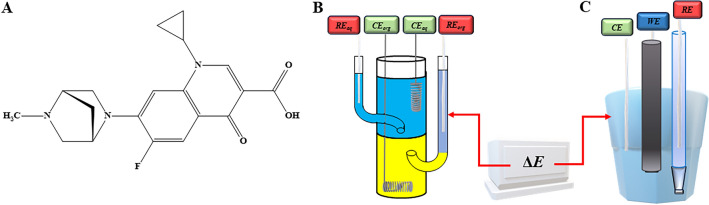
(**A**) The chemical structure of DANO. (**B**) The conventional ITIES glass cell with CE_aq_/CE_org_ (Pt, counter electrodes), and RE_aq_/RE_org_ (Ag/AgCl, reference electrodes) used for the aqueous and organic phase, respectively . (**C**) Traditional voltametric three-electrode setup equipped with CE – counter electrode (Pt), WE—working electrode (GCE) and RE—reference electrode (Ag/AgCl/3M KCl).

Danofloxacin (DANO, Fig. 1A) is a FQ with a broad spectrum of activity against Gram-negative and -positive bacteria. It is a synthetic drug specifically designed for veterinary applications and is primarily employed in the treatment of respiratory and gastrointestinal infections in animals^3^. The mechanism of action of DANO involves the inhibition of bacterial DNA gyrase and topoisomerase IV, which are enzymes for DNA replication and repair. Anticipated inhibition leads to the disruption of bacterial DNA synthesis and results in bacterial cell death. DANO exhibits good oral bioavailability and tissue penetration, making it suitable for both oral and parenteral administration. Its pharmacokinetic properties contribute to its effectiveness in treating systemic and localized infections in animals^4^. The safety profile of DANO is generally favorable, but like all FQs, it can be associated with adverse effects, including gastrointestinal disturbances, musculoskeletal malfunctioning and may negatively affect a central nervous system. It is important to use DANO judiciously and follow recommended dosing guidelines to minimize the risk of adverse reactions^5^^,^^6^. Overall, DANO plays a crucial role in veterinary medicine. However, its use should be guided by responsible antibiotic stewardship to preserve its efficacy and minimize the overdosing and development of antibiotic resistance. FQs structure, including DANO, facilitate the complex formation with multivalent cations, which is an interesting property further attracting the attention of scientists. It is expected, that complex will have different properties as compared to a free drug molecules^7,8^. We have found that this molecule was already electrochemically studied at the hanging mercury drop electrode (HMDE) based on the carbonyl group reduction signal^9^. DANO was determined mainly by chromatographic techniques such as high-performance liquid chromatography (HPLC) with UV detection^10^, ultra-high performance liquid chromatography-tandem mass spectrometry (UPLC-MS/MS) combined with solid phase extraction (SPE)^11^, QuEChERS technique with ultra-performance liquid chromatography–tandem mass spectrometry^12^ or electrophoretic techniques such as capillary electrophoresis (CE) coupled with laser-induced fluorescence (LIF) detection^13^.

Electrochemical techniques have emerged as powerful tools in diverse scientific disciplines due to their ability to investigate and control charge transfer processes at various interfaces^14–16^. These techniques encompass a wide range of methodologies, including voltammetry^14,17^, impedance spectroscopy^18,19^, potentiometry^20^ and many others^21^, each offering unique insights into electrochemical systems. In recent years, one of the dynamically developing branch of electrochemistry is based on the Interface Between Two Immiscible Electrolyte Solutions (ITIES)^22^. Detection at ITIES is usually beyond the analytical signals derived from the oxidation/reduction reactions; as the mechanism standing behind the recorded currents frequently involves the transfer of ionic species across the polarizable liquid–liquid interface (LLI). This sensing mechanism allows the detection of charged molecules that may remain electrochemically inert at solid electrodes (do not give the oxidation/reduction signals within available potential windows)^23^. The beneficial properties of the ITIES can be also harvested when the interfering species electrochemically active at the solid electrodes, do not undergo interfacial ion transfer reactions^24^^,^^25^. In the literature, several notable studies have been described, detailing experiments related to the investigation or quantification of FQs utilizing ITIES^6,26–28^. This research contributes to the development of the knowledge focused on the electroanalytical detection, and physicochemical studies focused on FQ antibiotics, which may find applications especially in the field of analytical chemistry and environmental science.

In this paper, we have employed two electroanalytical platforms to study DANO. First, is based on the four electrode configuration with soft and electrified junction formed between water and 1,2-dichloroethane solutions serving as the transducing element. The second is based on the traditional three electrode configuration with GCE used as the working electrode. With voltammetry we successfully determined a number of electroanalytical and physicochemical parameters that were then comparted (ITIES vs GCE) and evaluated. DANO was studied at the ITIES with ion transfer voltammetry (ITV). In turn, the electrochemical activity of DANO on the GCE was investigated with square wave voltammetry (SWV) and cyclic voltammetry (CV). The essential aspect of this paper involved the electrochemical detection of DANO from spiked milk samples. In this respect, we have used two chosen transducing elements with the optimized sensing conditions. The outcomes of these investigations are validated, whereas all analytical parameters such as limits of detections (LODs), limits of quantifications (LOQs), the linear dynamic ranges (LDRs) and detection sensitivities are presented in a comprehensive form.

## Materials and methods

### ITIES studies

**Scheme 1:**
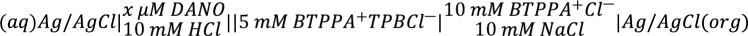
Electrochemical cell employed for electroanalytical studies of DANO at ITIES.

**Scheme 2:**
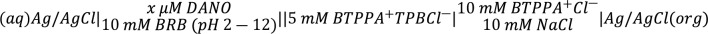
Electrochemical cell used to evaluate the effect of pH on the DANA behavior at ITIES.

All electroanalytical studies were conducted using an AUTOLAB-PGSTAT302N instrument (Metrohm Autolab B.V., The Netherlands) controlled via NOVA 1.11.1 software. Measurements were carried out with a four-electrode electrochemical system (Fig. 1B) comprising of two platinum (Pt) wires serving as the counter electrodes and two silver/silver chloride wires (Ag/AgCl) functioning as reference electrodes (as depicted in Scheme 1). The Galvani potential difference of the studied ions was calculated using the data from cyclic voltammograms whereas the potential axis was calibrated using the standard Galvani potential difference of the tetrapropylammonium cation ion transfer (TPrA^+^, ${\Delta }_{org}^{aq}{\Phi }_{{TPrA}^{+}}^{0}=-0.091 V$)^29^. Cells from Schemes 1 and 2 were used during this study.

### Measurements at the GCE

This part of measurements were conducted using an EmStat3 potentiostat under the control of PSTrace software supplied by PalmSens B.V., the Netherlands. All experiments were carried out in conjunction with an automated electrode stand (M164, MTM Anko Instruments, Cracow, Poland). The electrochemical three-electrode setup (Fig. 1C) consists of glassy carbon electrode (GCE) with a 3 mm diameter and a geometric area of 7.1 mm^2^ (Basi^®^, USA) as the working electrode, silver/silver chloride electrode (Ag/AgCl/3.0 mol L^−1^ KCl, MTM Anko Instruments, Kraków, Poland) as a reference electrode and a platinum wire (Pt, 99.99%, The Mint of Poland, Warsaw, Poland) as an auxiliary electrode. Square-wave voltammetry (SWV) and cyclic voltammetry (CV) were employed as the electroanalytical techniques to investigate DANO behavior at the GCE.

### Reagents and solutions

All chemicals utilized in this study were of analytical reagent grade. DANO with a purity of ≥ 98% was procured from Merck. Fresh stock solutions of DANO (10 mM) were meticulously prepared in graduated glass flasks by dissolving the calculated quantity of DANO in the appropriate volume of 10 mM hydrochloric acid (HCl) (studies at the ITIES) or deionized water (studies at the GCE). Potassium tetrakis(4-chlorophenyl)borate (KTPBCl, > 98%) and bis(triphenylphosphoranylidene) ammonium chloride (BTPPACl, 97%) were obtained from Merck and served as substrates for synthesizing bis(triphenylphosphoranylidene)ammonium tetrakis(4-chlorophenyl)borate (BTPPATPBCl) which was used as the background electrolyte in the organic phase. Tetrapropylammonium chloride (TPrACl, Alfa Aesar, > 99%,) served as the interfacially active internal reference probe used to calibrate the potential axis to the Galvani potential difference. The chemicals for the preparation of Britton–Robinson buffers (BRBs) were procured from POCH. A BRB matrix with a concentration of 40 mM was formulated by dissolving H_3_BO_3_, H_3_PO_4_, and CH_3_COOH in 10 mM NaCl (ITIES) or deionized water (GCE). The BRB matrix was titrated with 0.2 M NaOH to achieve the required pH values spanning through the range 2.0–12.0. 1,2–dichloroethane (1,2–DCE, POCH) and deionized water were employed as the solvents for the preparation of solutions in the organic and aqueous phases, respectively. Analytical standards for potentially interfering agents (citric acid, galactose, lactose, glucose, calcium chloride, potassium chloride, magnesium chloride, iron(III) chloride, sodium lactate and orthophosphoric acid (V)) were procured in their analytical reagent-grade from different suppliers (Sigma–Aldrich, Chempur, Fisher Chemical or Alfa Aesar). Fresh stock solutions of interfering species (10 mM) were prepared in a glass graduated flasks by dissolving the appropriate amount of chosen analyte in 10 mL of 10 mM HCl. Only in the case of orthophosphoric acid (V) deionized water was used as a solvent. Shortly after preparation all solutions were subjected to 10 min of sonification and were stored at 4.0 °C. The real samples were ultra high temperature (UHT) milk (1.5% fat content), which were obtained from a local supermarket. The milk samples were not subjected to any preparation process. The pH of the BRBs and the aqueous phase solutions was adjusted utilizing a combined pH electrode (Polilyte Lab, Hamilton, Switzerland).

## Results and discussion

### ITIES studies

**Figure 2:**
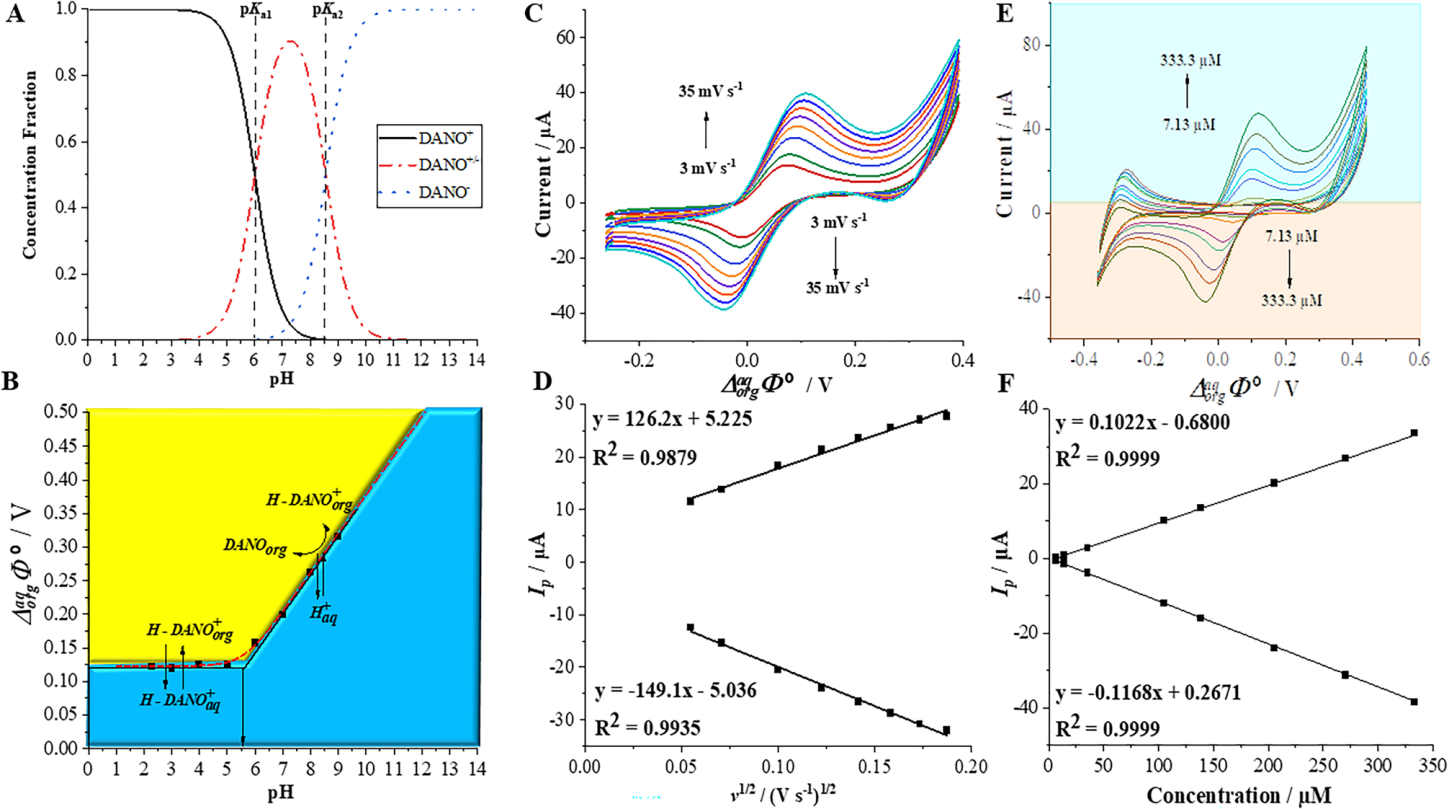
(**A**) Concentration fraction of different forms of DANO plotted in a function of the pH of the aqueous phase. p*K*_a1_ and p*K*_a2_ are marked with black dashed lines perpendicularly cutting the pH axis at their values. Existing DANO forms are presented in the figure legend. (**B**) Ion partitioning diagram plotted based on selected ITVs, recorded at different pH values of the aqueous phase. DANO and DANO^+^ correspond to the neutral and protonated form of analyte, respectively. (**C**) CV scan rate dependency for [DANO] = 0.33 mM recorded for increasing scan rates: 5; 10; 15; 20; 25; 30 and 35 mV s^−1^. (**D**) The corresponding plots representing the dependency between forward (positive) and backward (negative) current signals plotted in a function of the square root (v^1/2^) of the scan rate. (**E**) elected ITVs recorded for increasing DANO concentrations of 7.13; 14.24; 35.46; 104.9; 138.9; 205.5; 270.3 and 333.3 μM at pH = 2; scan rate 20 mV s^−1^. (**F**) The corresponding calibration curves showing the intensities of the forward (positive) and backward (negative) CV peak currents plotted in the function of the increasing DANO concentrations.

Initially, the comprehensive electroanalytical analysis of DANO was examined at the ITIES using ITV technique. The pH of the aqueous phase was adjusted to 2 being significantly lower than the DANO p*K*_a1_ and p*K*_a2_ values (6.07 and 8.50, respectively)^30^. Consequently, according to the analysis of the antibiotic’s structure and the concentration fraction diagram plotted for DANO (see Fig. 2A) all ionizable functional groups present within the studied analyte structure are protonated and hence the molecule is fully charged (exists in the aqueous phase as only cationic fraction). Figure 2B displays a graph depicting the DANO ion partition diagrams (dependency of the formal Galvani potential difference—${\Delta }_{org}^{aq}\Phi$—of the DANO ion transfer plotted in function of the aqueous phase pH). ${\Delta }_{org}^{aq}\Phi$ is taken from ITVs recorded in a broad pH range (2–12) of BRBs used as the aqueous phase, as illustrated in Fig. S1 (see electronic supporting information). In the structure of DANO we can distinguish a carboxylic group with p*K*_a1_ of approximately 6 and peripheral nitrogen atoms, which are part of the piperazine ring with pK_a2_ value around 8.5. With this in mind, at pH 2, the carboxylic acid groups are not dissociated, and the piperazine units are protonated, leading to a fully positively charged antibiotic molecule. To transfer the positively charged (cationic) molecules from the aqueous phase to the organic phase, the LLI was polarized from less positive to more positive potentials during the forward scan^6^. When the pH of the aqueous phase is significantly lower than the DANO *pK*_*a*_ value (pH 5), the positively charged analyte undergoes a direct ion transfer reaction from the aqueous to the organic phase upon application of a Galvani potential difference exceeding + 0.123 V. Since the DANO possess two functionalities that can be either positively or negatively charged, in the pH range from 5 to 10 a fraction of zwitterions exists in the aqueous phase with a peak concentration found at around 7. With an increase in the pH of the aqueous phase, neutral/zwitterionic DANO molecules distribute into the organic phase (denoted by the vertical black arrow pointing towards x axis in Fig. 2B). The presence of neutral DANO in the organic phase can facilitate the transfer of protons from the aqueous to the organic phase, necessitating Galvani potential difference values exceeding + 0.123 V. The expected behavior of DANO is depicted by the dashed red line calculated according to Eq. 1 which is in line with the experimental findings marked on the Fig. 2B with black data points .

1 $${\Delta }_{org}^{aq}{\phi }_{0.5}={\Delta }_{org}^{aq}{\phi }^{0}+\frac{RT}{nF}\text{ln}(\frac{{10}^{-pH}+{K}_{a}{K}_{D}+{K}_{a}}{{10}^{-pH}})$$

In Eq. 1 the acid dissociation constant is represented as *K*_a_ (p*K*_a_ value of 8.50). K_D_ is the distribution constant, delineating the ratio between the concentration of the non-protonated form of DANO present in the aqueous [DANO]_aq_ and the organic phase [DANO]_org_:

2 $${K}_{D}=\frac{{[DANO]}_{aq}}{{[DANO]}_{org}}$$

The experimental results demonstrated that the optimal correlation was obtained for a *K*_*D*_ value of around 600, suggesting the inherently hydrophobic characteristics of DANO molecules in the neutral form. In more accessible terms, for every 600 molecules of DANO in the organic phase, 1 molecule will be present in the aqueous phase when the pH approaches the p*K*_a2_ value.

Figure 2C shows a series of ITVs recorded for fixed concentration of DANO (333.3 μM) and varied potential scan rate value. The observed process demonstrated reversibility, with the forward and backward currents intensity ratio being close to unity, while the peak-to-peak separation (Δ*E*_*p*_) was found to be ~ 75 mV (measured for ITV recorded at 25 mV s^−1^). This value is in proximity to the anticipated theoretical value of 59 mV z^−1^ (z = 1), indicating the mono-charged nature of the DANO cation undergoing ion transfer reaction. Deviation from the expected theoretical value of 59 mV z^−1^, where z represents the molecular charge of the analyte, is commonly observed for polarized LLIs and arises from the resistive properties of the organic phase^6,31^. By analyzing the linear fit equation of the relationship between the current signal and the square root of the scan rate (*v*^1/2^) (Fig. 2D) and the Randles–Ševčík equation we have calculated the aqueous and the organic DANO diffusion coefficients (*D*). Obtained values were equal to *D*_*aq→org*_ = 1.13 × 10^–6^ cm^2^ s^−1^ and *D*_*org→aq*_ = 0.14 × 10^–6^ cm^2^ s^−1^. Another parameter derived from ITVs is the formal Galvani potential of DANO (in cationic form) ion transfer ($\Delta _{{org}}^{{aq}} \Phi ^{\prime }$). This parameter is closely related to the hydrophobicity/-philicity of the analyte under study. For cationic species, a higher value of $\Delta _{{org}}^{{aq}} \Phi ^{\prime }$ signifies greater hydrophilicity^32^. The $\Delta _{{org}}^{{aq}} \Phi ^{\prime }$ serves as a valuable parameter, which, in conjunction with Eq. 3, enables the calculation of the formal water | 1,2-DCE partition coefficient ($logP_{{water/DCE}} ^{\prime }$). This coefficient quantitatively characterizes the partitioning behavior of the charged molecule between the aqueous and the organic (1,2-DCE) phases^33^.

3 $$logP_{{water/DCE}} ^{\prime } = - \frac{{\Delta _{{org}}^{{aq}} \Phi ^{\prime } z_{i} F}}{{2.303RT}}$$

where: $\Delta _{{org}}^{{aq}} \Phi ^{\prime }$ is the formal Galvani potential of the ion transfer reaction (V); *z*_*i*_—charge of the investigated analyte; *F*—the Faraday constant (96,485 C·mol^−1^); *R*—the gas constant (8.314 J mol^−1^ K^−1^) and *T*—the temperature (298 K). For DANO, the calculated $logP_{{water/DCE}} ^{\prime }$ is − 2.08, indicating its relatively high hydrophilicity (given that it is built from the aromatic rings and has fluorine substituent). This value is also in line with ${logP}_{water/octanol}$ = − 1.37 (pH 3) reported by G.M. Cardenas-Youngs and J.L. Beltrán^34^ which also suggests that DANO is a hydrophilic compound. Ultimately, the $\Delta _{{org}}^{{aq}} \Phi ^{\prime }$ was employed to calculate the formal Gibbs free Energy of the interfacial ion transfer reaction ($\Delta {G}^{{\prime}, aq\to org})$ according to Eq. 4:

4 $$\Delta G^{{\prime ,aq \to org}} = z_{i} F\Delta _{{org}}^{{aq}} \Phi ^{\prime }$$

All physicochemical parameters determined for DANO are presented in Table S1 in electronic supporting information.

**Figure 3:**
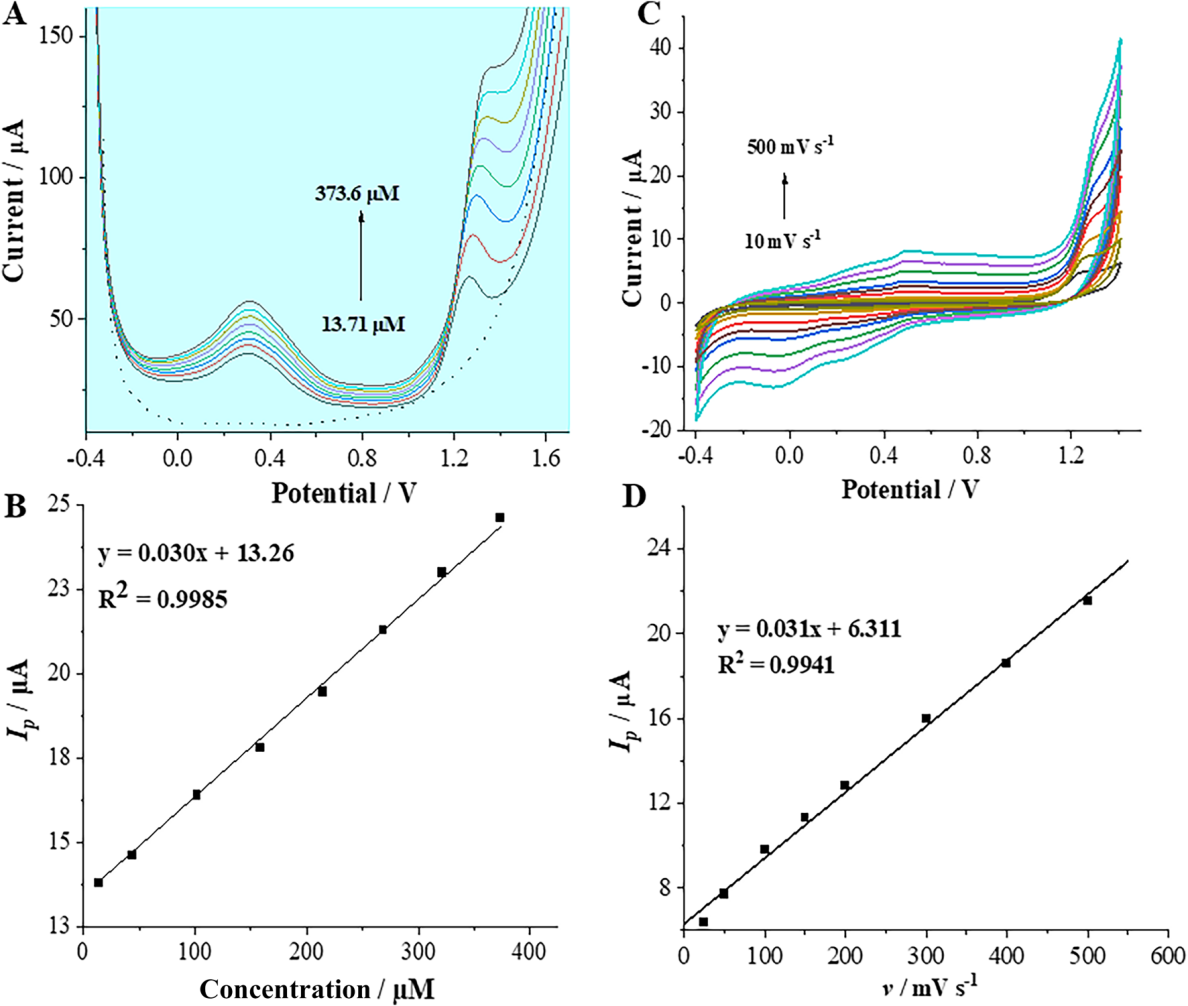
(**A**) Selected SWVs recorded for increasing DANO concentrations of 13.71; 43.37; 101.6; 158.6; 214.2; 268.5; 321.6 and 373.6 μM together with blank (dashed line), recorded in BRB, pH = 2, optimal conditions: *f* = 60 Hz, *E*_*SW*_ 90 mV and Δ*E* = 12 mV. (**B**) The corresponding calibration curve of DANO (Δ*E* = 0.25 V). (**C**) CVs of 0.15 mM DANO recorded at scan rates in the range from 10 to 500 mV s^−1^ in BRB (pH 2.0). (**D**) The plot of the peak current (*I*_*p*_) versus the scan rate (*v*).

Finally, we harvested the fact that the DANO is electrochemically active at the ITIES to developed the procedure for the electroanalytical determination of the concerned analyte. For this purpose, the ITVs were recorded for increasing DANO concentrations, as illustrated in Fig. 2E. Subsequently, the dependencies of forward and backward peak current intensities *vs*. *C*_DANO_ were plotted, as shown in Fig. 2F. Notably, in both cases, DANO transfer from the aqueous to the organic phase and vice versa, the coefficients of determination (R^2^) approached unity. The linear relationship between *Ip* values (positive or negative currents) and *C*_*DANO*_ within the LDR of 7.13–333.3 µM is evident from Fig. 3F. Based on these findings, crucial electroanalytical parameters including linearity, sensitivity, LODs, LOQs, were determined and are compiled in Table 1.

**Table 1:** Electroanalytical parameters of DANO obtained at the ITIES and GCE.

Parameter	Employed configuration	
	ITIES	GCE
Number of repetitions	3	3
Linear concentration range [μM]	7.13–333.3	13.71–373.6
Slope (*a*) (A M^−1^)	0.1022 ^aq→org^ 0.1168 ^org→aq^	0.0300
Normalized Slope (*a*) (A M^−1^ cm^−2^)^a^	0.0768 ^aq→org^ 0.0878 ^org→aq^	0.4225
Standard error of slope (*SE*_*a*_) (A M^−1^)^b^	0.0004 ^aq→org^ 0.0004 ^org→aq^	0.0005
Intercept (*b*) (μA)	0.1022 ^aq→org^ − 0.1168 ^org→aq^	0.0300
Standard error of intercept (*SE*_*b*_) (μA)^b^	0.0725 ^aq→org^ 0.0787 ^org→aq^	0.1037
Coefficient of determination (*R*^2^)	0.9999 ^aq→org^	0.9986
	0.9999 ^org→aq^	
LOD (μM)^c^	2.13 ^aq→org^	10.37
	2.02 ^org→aq^	
LOQ (μM)^d^	7.09 ^aq→org^	34.53
	6.74 ^org→aq^	

^a^Slope values divided by the geometric surfaces of LLI and GCE, respectively.

^b^*SE* = *SD/n*^1/2^.

^c^LOD = 3SD_b_/a.

^d^LOQ = 10SD_b_/a; a—slope and b—intercept; aq → org—corresponds to parameter calculated for the positive signals; org → aq—corresponds to parameter calculated for the negative signals.

### Electroanalytical study of DANO at GCE

The next step of this work involved utilizing GCE combined with SWV and CV techniques for the electrochemical investigation and determination of DANO. The electrochemical response of DANO was first investigated across wide pH spectrum provided by BRB (pH 2–10, Fig. S2A). The SWV studies were carried out within the potential range from + 0.4 V to + 1.5 V. Preliminary analysis revealed that DANO exhibits two oxidation signals, one at approximately + 0.25 V and the other at around + 1.2 V versus Ag/AgCl (3 M KCl). The analytical signal with a better-defined shape and higher current intensity was observed at the potential ~  + 0.25 V. Therefore, for the purpose of this study, this signal was subjected to further investigations and electroanalytical quantification of DANO. The most pronounced signals of DANO were detected under acidic conditions (pH 2.0), hence the BRB with pH = 2 was chosen as the supporting electrolyte for subsequent studies (Fig. S2B from electronic supporting information). Additionally, we have noticed that as the pH increased, the oxidation peak of DANO shifted towards more cathodic potentials, indicating the involvement (as expected) of protons in the electrochemical process^35^. Furthermore, the relationships between the peak potential (*E*_*p*_) and pH is linear only in pH range (3–7) (Fig. S2C), suggesting that the electrochemical reaction is more complex, and depends on factors other than hydrogen or hydroxyl ions concentration in the solution.

To scientifically assess the optimum conditions for determining DANO using SWV in conjunction with GCE, the influence of potential modulation parameters, such as frequency (*f*), amplitude (*E*_*SW*_) and step potential (*∆E*) were examined. The obtained results, indicated that the highest oxidation peak (at approximately + 0.25 V) and the best shape of the DANO signal were observed with the following parameters: a frequency of 60 Hz, an amplitude of 90 mV and a step potential of 12 mV. Finally, the developed SWV procedure was employed for DANO determination in a model sample. SWV technique was used under the optimal experimental conditions. The usefulness of the SWV for the assay of DANO was estimated as a function of the peak current (*I*_*p*_) of increasing DANO concentrations (*C*_*DANO*_) in three runs (n = 3). The developed procedure for SWV determination of DANO was also validated. The significant validation parameters, such as linearity, LOD, LOQ and precision were evaluated (see Table 1). The SWVs and the corresponding calibration graph are depicted in Fig. 3. As can be noticed from this figure, the oxidation peak current increased linearly in LDR of 13.71 to 373.6 μM.

The CV technique facilitates the extraction of valuable insights regarding the electrode process, including kinetic parameters, reversibility or its nature (diffusion- or adsorption controlled charge transfer processes)^36^. In this paper CV technique was employed to elucidate the electrochemical behaviour of DANO. CV analyses of DANO were carried out within a potential range from -0.4 to 1.40 V, at scan rates in the range of 10–500 mV s^−1^. The recorded CVs, conducted in the presence of [DANO] = 0.15 mM in BRB solution at pH 2, revealed an electrochemical process spanning nearly over entire potential window (from − 0.2 V to 0.9 V) and only one analytical, anodic peak at approximately + 1.30 V, which was analysable (Fig. 3C). The latter most probably originated from the oxidation of the peripheric nitrogen atom from the piperazine ring. To assess the nature of the electrochemical process happening at the GCE during DANO oxidation, the dependence of the *I*_*p*_ on the *v* was analyzed (Fig. 3D). The relationship *I*_*p*_* vs*. *v* shows a linear correlation, which indicates an adsorption-controlled process. To confirm the obtained results, a plot correlating the logarithms of the peak current (log *I*_*p*_) and the scan rate (log *v*) was generated, yielding a slope of 0.40 (R^2^ = 0.9798) (Fig. S3), closely aligning with the theoretically anticipated value of 0.5 for an diffusion-controlled process^36,37^. Hence, in this case the character of DANO oxidation process at the GCE is not unequivocal and indicated a mixed adsorption-diffusion process^36,38,39^.

### Real samples analysis

The next stage of this research involved the application of both developed procedures (ITV and SWV at ITIES and GCE, respectively) for the DANO determination in samples of cow’s milk. The samples analyzed comprised ultra-high temperature (UHT) milk with a fat content of 1.5% obtained from a nearby supermarket.

#### DANO detection at ITIES

**Figure 4:**
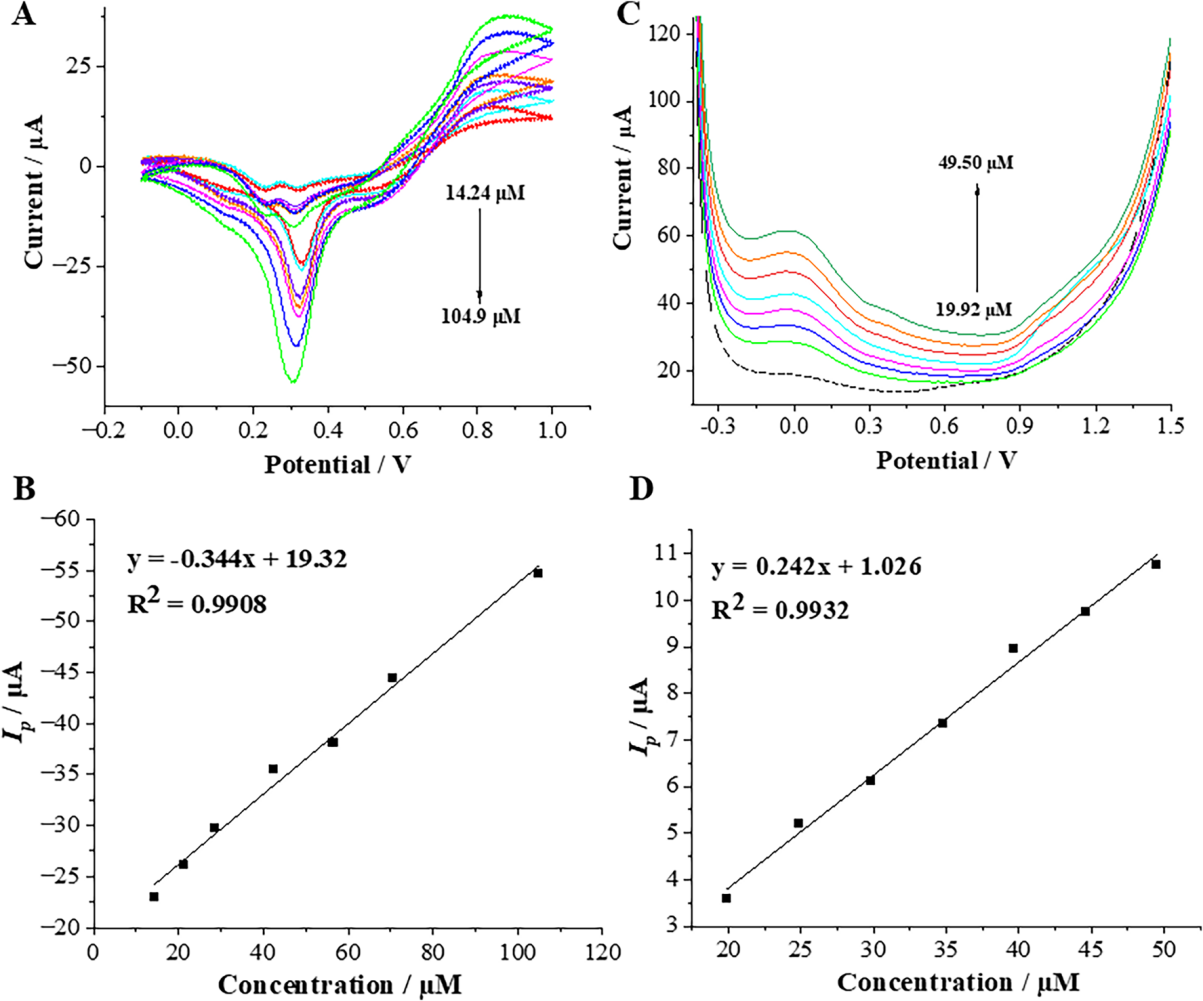
(**A**) ITVs recorded for increasing DANO concentrations added to milk sample (after subtracting the blank) for increasing DANO concentrations of 14.24, 21.34, 28.41, 42.49, 56.50, 70.42 and 104.9 µM, conditions: *v* = 20 mV s^−1^. (**B**) The corresponding calibration curve of DANO determination in milk samples at ITIES. (**C**) SWVs recorded for increasing DANO concentrations added to milk sample for increasing DANO concentrations of 19.92, 24.88, 29.82, 34.76, 39.68, 44.60 and 49.50 µM together with blank (dashed line), optimal conditions: *f* = 60 Hz, *E*_*SW*_ 90 mV and Δ*E* = 12 mV. (**D**) The corresponding calibration curve of DANO determination in milk samples at GCE.

In ITIES-based experiments, the aqueous phase was substituted with a 3.5 mL of the milk sample, which did not necessitate prior purification (or treatment) to eliminate fats, proteins, saccharides, or other chemical constituents. Subsequently, suitable volumes of DANO standard solution were introduced into the test sample, and the ITVs were recorded in three runs (n = 3) as the DANO concentrations in the milk sample increased. Figure 4A depicts the ITVs obtained during the addition of specific volumes of DANO stock solution into the milk samples, after subtracting blank reading (recorded in the absence of DANO). The analytical signals, manifested as negative currents, displayed a linear correlation (Fig. 4B) with the increasing DANO concentration within the LDR of 14.24 to 104.9 µM. The LODs were derived from the calibration curves. The calculated LOD and LOQ values (7.32 and 24.38 µM, respectively) for the determination of DANO in milk samples was determined based on the signals attributed to the analyte transfer from the organic to the aqueous phase.

#### DANO detection at GCE

To determine DANO, we have started by recoding the SWV at GCE immersed into 10 mL of milk sample (used instead of supporting electrolyte). Electroanalytical parameters applied at this stage were taken from the optimization study described in section Electroanalytical study of DANO at GCE. Following that, the standard addition method was employed. Consecutive volumes of DANO stock solutions were introduced into the voltammetric cell utilizing a micropipette. The SWVs together with corresponding calibration graph are depicted in Fig. 4C, D. As can be noticed from Fig. 4C the oxidation peak current of DANO increased linearly within the LDR of 19.92 to 49.50 µM. The calculated LOD and LOQ values for the determination of DANO in milk samples was determined to be 4.00 and 13.32 µM, respectively.

#### Interference studies

One prevalent challenge in chemical analysis involves the impact of interfering agents (*IA*) on the recorded analytical signals. We can only claim that the method is selective when the impact of the interfering species do not exceed ± 10%^40^. Despite the fact that both developed procedures exhibited high applicability, as they allowed for the determination of DANO in such a complex matrix as milk samples without difficulty, the impact of potential interfering species on the recorded DANO signals was also investigated. Consequently, the influence of potential interferents, including milk contaminants, such as: citric acid, galactose, lactose, glucose, calcium cations, potassium cations, magnesium cations, iron(III) cations, sodium lactate and orthophosphate (V) anions was evaluated by means of both elaborated procedures.

#### DANO detection at ITIES

Initially, ITVs were recorded for [DANO] = 70.40 µM, which served as a reference. Subsequently, the appropriate amount of interferent standard solution was added to the aqueous phase placed in the ITIES cell to achieve concentrations 14.3, 140.6, 277.4 and 1248.0 µM, respectively. After each aliquot of interfering agent (IA) addition the ITVs were recorded. Based on the results, it was observed that only in the case of iron (III) ions and citric acid, their significant influence on the recorded DANO signals was observed for each studied concentration. For the remaining *IA,* only at the highest *IA* concentration (1248 µM), their effect on the recorded DANO signals was observed. For the other *IA* concentration in the range from 14.3 to 277.4 µM, their influence on the analyte signals did not exceed 5.4%. Detailed information regarding the impact of potential *IA* is presented in Table S2 from electronic supporting information.

#### DANO detection at GCE

The interference study started with voltametric analysis of 10 mL of the supporting electrolyte placed in the voltammetric cell (BRB, pH = 2), followed by the addition of 143 µL of the DANO stock solution (*C*_*DANO*_ in cell = 62.55 µM). Subsequently, the SWV of DANO was recorded. Then, specific volumes of *IA* stock solutions were added to the cell containing DANO at concentrations: 6.09, 56.20 and 572.4 µM, and voltammograms were recorded after each addition of *IA*. Unfortunately, the results of the experiments conducted at the GCE indicate low selectivity of the developed method. For all investigated *IA,* their significant influence on the recorded DANO signals was observed. Only in the case of potassium cations, magnesium cations, citric acid, sodium lactate and orthophosphate (V) anions, at their lowest concentration (6.09 µM), the influence of *IA* on the DANO signals did not exceed 10% (see Table S2 from electronic supporting information).

## Conclusions

This study compares the electroanalytical capabilities of two systems: (1) first based on GCE used as the sensing element and (2) second, on the ITIES, for the detection of the fluoroquinolone antibiotic–danofloxacin. The objective of this work was to determine the optimal conditions for detecting DANO using both systems and derive various electroanalytical and physicochemical parameters pertaining to the studied analyte. Additionally, the detection of DANO was carried out in spiked milk samples by means of standard addition method. To the best of our knowledge, this study represents the first attempt to directly compare electroanalytical parameters obtained with a solid electrode and eLLI. The analysis of the obtained results revealed that both developed procedures are useful in electrochemical investigation and determination of DANO. Nevertheless, based on the obtained results, there is a clear advantage of the system based on the ITIES compared to the system based on GCE (even though for the later we have used more sensitive electroanalytical methodology, this is SWV). ITIES based platform features better electroanalytical parameters (lower LOD and LOQ values). We have also found that ITIES based platform was not affected by the interfering species that can be found in milk samples as much as the redox signals originating from the DANO oxidation at the GCE.

### Supplementary Information

Supplementary Information.

## Data Availability

The datasets generated and/or analysed during the current study are available in the ZENODO repository, https://zenodo.org/records/10813557.
