# Supplementary material for: Comparative electrochemical study of veterinary drug danofloxacin at glassy carbon electrode and electrified liquid–liquid interface
Source: Sci Rep. 2024 Jun 24;14:14489. doi: 10.1038/s41598-024-65246-3 (PMC11196252; doi:10.1038/s41598-024-65246-3)
Supplement: Supplementary file 1 — Supplementary Information. [file 41598_2024_65246_MOESM1_ESM.docx]

**Comparative electrochemical study of veterinary drug danofloxacin at glassy carbon electrode and electrified liquid-liquid interface.**

Konrad Rudnicki*^a^, Sylwia Budzyńska ^b^, Sławomira Skrzypek ^a^, Lukasz Poltorak** ^a^

^a^ University of Łódź, Faculty of Chemistry, Department of Inorganic and Analytical Chemistry, Tamka 12, 91-403 Łódź, Poland

^b^ Poznań University of Life Sciences, Faculty of Forestry and Wood Technology, Department of Chemistry, Wojska Polskiego 75, 60-625 Poznań, Poland

First corresponding author: konrad.rudnicki@chemia.uni.lodz.pl

Second corresponding author: lukasz.poltorak@chemia.uni.lodz.pl

***Abstract***

This work compares the electroanalytical performance of two electroanalytical systems based on (i) the glassy carbon electrode (GCE), and (ii) the electrified liquid-liquid interface (eLLI), for the detection of fluoroquinolone antibiotic – danofloxacin (DANO). Our aim was to define the optimal conditions to detect the chosen analyte with two employed systems, extract a number of electroanalytical parameters, study the mechanism of the charge transfer reactions (oxidation at GCE and ion transfer across the eLLI), and to provide physicochemical constants for DANO. Detection of the chosen analyte was also performed in the spiked milk samples. To the best of our knowledge, this is the first work that directly compares the electroanalytical parameters obtained with solid electrode (in this case GCE) and eLLI. We have found that for DANO the latter provides better electroanalytical parameters (lower LOD and LOQ) as well as good selectivity when the milk was analyzed.

**Table of contents**

Figure S1 page 3

Table S1 page 4

Figure S2 page 4

Figure S3 page 4

Figure S4 page 5

Table S2 page 6

**Fig. S1.** The effect of pH of the aqueous phase on the electrochemical behaviour of DANO recorded in BRB solutions within the pH range 2-12. Conditions: Technique: CV; *v* = 10 mV s^−1^; [DANO] = [TPrA^+^] = 84.99 µM.

**Table S1.** Selected physicochemical and electroanalytical parameters for DANO.

| Analyte | z | ${pKa}_{1}$ | ${pKa}_{2}$ | D [cm^2^ s^-1^]_aq→org_ ^a^ | D [cm^2^ s^-1^] _org→aq_ ^a^ | ${logP}_{water/octanol}$ | ${logP}_{water/DCE}^{'}$ ^b^ | $\Delta_{org}^{aq}\Phi^{'}$ [mV] | $\Delta G^{', aq\to org}$ [kJ mol^-1^] ^c^ |
| --- | --- | --- | --- | --- | --- | --- | --- | --- | --- |
| *DANO* | 1 | 6.07[29] | 8.56[29] | 1.13×10^-6^ | 1.44×10^-7^ | -1.37[33] | -2.08 | 123 | 11.87 |

^a^ calculated from the Randles – Ševčík equation.
^b^ see eq. 3
^c^ see eq. 4


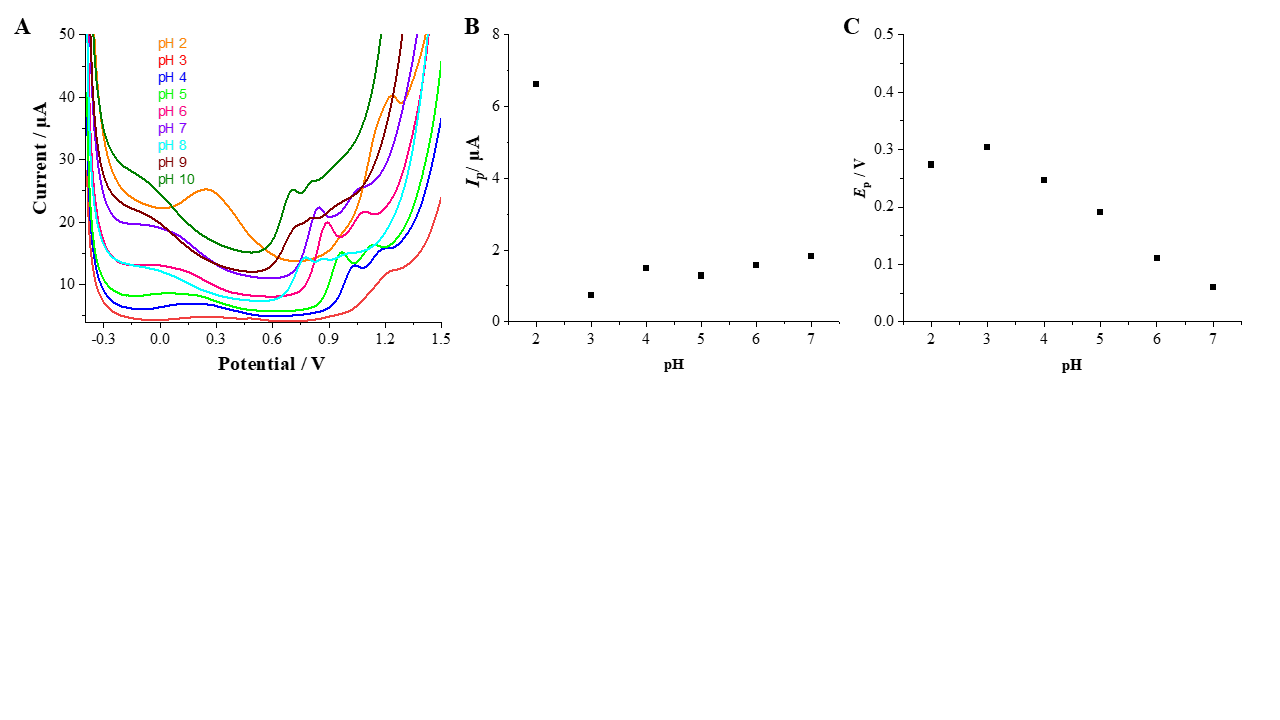


**Fig. S2.** A – The effect of pH of supporting electrolyte on the SWV behaviour of [DANO] = 15.00 µM, recorded on the GCE in BRBs. B – The plot of SWV peak currents (*I_p_*) *vs*. pH. C – The plot of peak potentials (*E_p_*) *vs*. pH. Conditions: *E_SW_ =* 100 mV; *f* = 40 Hz; Δ*E* = 10 mV.


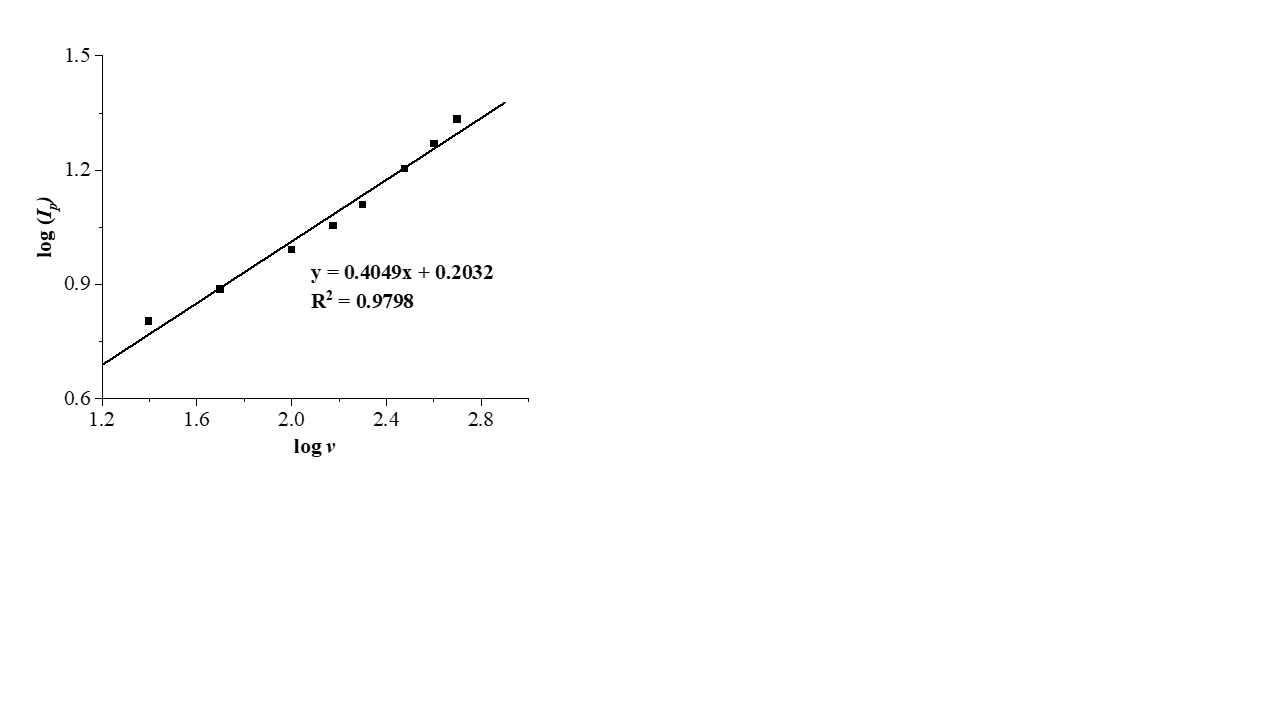


**Fig. S3.** The dependence of log *I_p_ vs.* log *v* for DANO at GCE*.* The supporting electrolyte was BRB solution, pH 2. Results based on the CV technique measurements recorded at scan rates in the range from 10–500 mV s^−1^.

 **Fig. S4.** ITVs recorded for increasing DANO concentrations added to milk sample for increasing DANO concentrations of 14.24, 21.34, 28.41, 42.49, 56.50, 70.42 and 104.9 µM. Conditions: *v* = 20 mV s^-1^.

**Table S2.** The percentage impact of each individual interferring agent on the recorded DANO signals was provided. Green cells indicate negligible, acceptable influence for selective methods, while red cells denote unacceptable influence for a selective method. ↑ - signal increase, ↓ - signal decrease.

| Procedure  Interfering agent | ITIES | | | | GCE | | |
| --- | --- | --- | --- | --- | --- | --- | --- |
| *c_DANO_* [μM] | 70.40 | | | | 62.55 | | |
| Fe^3+^ | 25 ↓ | 193 ↑ | 214 ↑ | 221 ↑ | 28 ↑ | 29 ↑ | 37 ↑ |
| Mg^2+^ | 1.7 ↓ | 2.5 ↓ | 3.3 ↓ | 28 ↓ | 0 | 16 ↓ | 50 ↓ |
| Ca^2+^ | 1.7 ↑ | 3.4 ↑ | 3.4 ↑ | 0.9 ↓ | 21 ↓ | 34 ↓ | 42 ↓ |
| K^+^ | 1.7 ↑ | 0.9 ↑ | 0.9 ↑ | 14 ↓ | 5 ↓ | 14 ↓ | 24 ↓ |
| Citric acid | 19.5 ↓ | 20 ↓ | 28 ↓ | 37 ↓ | 9 ↓ | 39 ↓ | 45.5 ↓ |
| Lactose | 0 | 1.7 ↑ | 3.3 ↑ | 7 ↓ | 11 ↓ | 19 ↓ | 33 ↓ |
| Sodium lactate | 2.5 ↓ | 3.2 ↓ | 3.8 ↓ | 19 ↓ | 1 ↑ | 21 ↑ | 29 ↑ |
| Glucose | 2.7 ↑ | 4.1 ↑ | 5.4 ↑ | 6.8 ↓ | 33 ↓ | 36 ↓ | 38 ↓ |
| Galactose | 2.7 ↑ | 4.1 ↑ | 5.4 ↑ | 9 ↓ | 126 ↑ | 133 ↑ | 21 ↑ |
| Ortophosphoric (V) acid | 1.3 ↓ | 2.5 ↓ | 3.9 ↓ | 76 ↓ | 5 ↓ | 19 ↓ | 40.5 ↓ |
